# The Smaller the Leaf Is, the Faster the Leaf Water Loses in a Temperate Forest

**DOI:** 10.3389/fpls.2019.00058

**Published:** 2019-02-04

**Authors:** Cunguo Wang, Junming He, Tian-Hong Zhao, Ying Cao, Guojiao Wang, Bei Sun, Xuefei Yan, Wei Guo, Mai-He Li

**Affiliations:** ^1^College of Agronomy, Shenyang Agricultural University, Shenyang, China; ^2^School of Geographical Sciences, Northeast Normal University, Changchun, China; ^3^Swiss Federal Institute for Forest, Snow and Landscape Research (WSL), Birmensdorf, Switzerland

**Keywords:** leaf area, leaf intensity, leaf mass, leaf size, leaf water loss, right-skewed distribution, trade-off

## Abstract

Leaf size (i.e., leaf surface area and leaf dry mass) profoundly affects a variety of biological carbon, water and energy processes. Therefore, the remarkable variability in individual leaf size and its trade-off with total leaf number in a plant have particularly important implications for understanding the adaption strategy of plants to environmental changes. The various leaf sizes of plants growing in the same habitat are expected to have distinct abilities of thermal regulation influencing leaf water loss and shedding heat. Here, we sampled 16 tree species co-occurring in a temperate forest in northeastern China to quantify the variation of leaf, stomata and twigs traits, and to determine the relationships of leaf size with leaf number and leaf water loss. We examined the right-skewed distributions of leaf size, leafing intensity, stomatal size and stomatal density across species. Leafing intensity was significantly negatively correlated with leaf size, accounting for 4 and 12% of variation in leaf area and leaf mass, respectively. Species was the most important factor in explaining the variation in leaf size (conditional *R*^2^ of 0.92 for leaf area and 0.82 for leaf mass). Leaf area and mass significantly increased with increasing diameter of twigs. Leaf water loss was strongly negatively correlated with leaf area and leaf mass during the first four hours of the measurement. Leaf area and leaf mass accounted for 38 and 30% of variation in total leaf water loss, respectively. Leaf water loss rate (*k*) was significantly different among tree species and markedly linearly decreased with increasing leaf area and leaf mass for simple-leaved tree species. In conclusion, the existence of a cross-species trade-off between the size of individual leaves and the number of leaves per yearly twig unit was confirmed in that temperate forest. There was strongly negative correlation between leaf water loss and leaf size across tree species, which provides evidences for leaf size in leaf temperature regulation in dry environment with strong radiation. The size-dependent leaf water relation is of central importance to recognize the functional role of leaf size in a changing climate including rapid changes in air temperature and rainfall.

## Introduction

Plants are certainly modular organisms with recognized capabilities to regulate size and number of organs at the module scale (Kroon et al., 2005). Leaves are the principal photosynthetic organs of plants (Wright et al., 2004), therefore, the size of leaves (e.g., leaf surface area, leaf dry mass and leaf length) profoundly affects a variety of biological processes, for instance, plant growth, survival, reproduction, and ecosystem function (Koch et al., 2004; Tozer et al., 2015). Thus, considerable attention has been paid to the natural variations in leaf size and its ecological and evolutionary significances (Niinemets et al., 2007). For example, leaf surface area varies over six orders of magnitude across terrestrial plants (Milla and Reich, 2007; Niinemets et al., 2007), and there is a 100-fold variation in leaf dry mass within a single climatic region (Kleiman and Aarssen, 2007). Leaf length in angiosperm trees varies from a few millimeter to over one meter with more than three orders of magnitude (Jensen and Zwieniecki, 2013). Considerable variations of leaf size among species are attributed to a wide range of plant traits including morphological and physiological characteristics and leaf energy balance (Westoby and Wright, 2003; Pickup et al., 2005; Niinemets et al., 2006).

Recently, the leaf size variation has been interpreted as the trade-off between leaf size and the number of leaves produced (Kleiman and Aarssen, 2007; Yang et al., 2008; Whitman and Aarssen, 2010). Leaf size across species was linearly negatively correlated with leafing intensity, the number of leaves per unit volume/mass of the twigs on which the leaves were borne (Kleiman and Aarssen, 2007; Ogawa, 2008; Yang et al., 2008; Huang et al., 2015). Consequently, a “leafing intensity premium” hypothesis at the twig level was proposed by Kleiman and Aarssen (2007). According to Kleiman and Aarssen (2007), the fitness benefits of higher leafing intensity (namely small leaves) are primarily associated with the fitness benefits of a larger pool of axillary buds, which in turn provide greater facility for wide phenotypic plasticity in the allocation of these meristems to vegetative *versus* reproductive functions (Kleiman and Aarssen, 2007). Based on the leaf size-number trade-off theory, it was inferred that leaf biomass density per unit twig volume was constant ranging from the twig to the canopy level in fully closed forest stands (Ogawa, 2008). Hence, the leaf size-number trade-off may have particularly important implications for understanding leaf size evolution, because it is one of the fundamental adaptation strategies of plants to environmental changes (Yang et al., 2008). Trees having small *versus* large leaves can show distinct leaf deployment strategies along a leaf size-number trade-off continuum (Scott and Aarssen, 2012). For instance, smaller leaves with higher density of major vein were more tolerant to vein embolism (Scoffoni et al., 2011).

Several previous studies have found that the remarkable variability in leaf size plays a prominent role in leaf thermal regulation (Givnish and Vermeij, 1976; Ackerly et al., 2002; Jensen and Zwieniecki, 2013; Wright et al., 2017). Leaf area can regulate leaf temperature via the thickness of leaf boundary layer (Ackerly et al., 2002; Niinemets et al., 2006), where heat transfer is slower relative to the more turbulent air beyond the leaf (Givnish and Vermeij, 1976; Jensen and Zwieniecki, 2013). The thickness of leaf boundary layer increases with increasing leaf area, so that the rate of heat convection per unit leaf area is greater between leaf and air for a small leaf than for a large leaf (Leigh et al., 2017). Smaller leaves are expected to have lower leaf temperatures than large leaves at sunny habitats, and thus to avoid overheating (Niinemets and Kull, 1994). Furthermore, leaf size tends to decrease with decreasing water availability (Mcdonald et al., 2003; Basal et al., 2005; Cramer et al., 2009). Generally smaller leaves are advantageous in hot and dry environments and at high intensities of solar radiation, while large leaves with less efficient energy exchange capacity are advantageous in cooler, moister and lower irradiance environments (Niinemets et al., 2006; Meier and Leuschner, 2008; Tozer et al., 2015).

A great deal of the variability in leaf size contributes to water balance. Because plant leaf is a critical component in the plant water transport system, accounting for 30% or more of whole-plant hydraulic resistance, especially in dry environments (Sack and Holbrook, 2006). Parameters (e.g., leaf water loss and initial leaf water content) measured on excised leaves at minimum stomatal aperture have been proposed as simple but reliable indicators of drought resistance in wheat, cotton, and sorghum (Hall and Jones, 1961; Basal et al., 2005), as well as forest species in northern China (Zhang and Li, 1995). Either high irradiation or extremely negative atmospheric water potential or both will lead to severe water stress and thus stomata closure of plants (Clarke et al., 1991; Cramer et al., 2009). Therefore, water supply to a leaf may depend on rates of cuticular transpiration (Schreiber and Riederer, 1996) if water required is sufficient. Efficient cuticular transpiration is also of great importance in order to provide sufficient nutrients to leaves, because nutrients are mainly transported with water from soil to leaves via the xylem (Yates et al., 2010).

Climate models have indicated that drought episodes will become more frequently because of global warming (Salinger et al., 2005). For example, severe drought events and daily temperature extremes have been revealed to become more frequent and widespread in northeastern China (Yu et al., 2014; Yu and Li, 2015). This emphasizes the urgent need to study the morphological and physiological adaptation strategies of plants to environmental changes including future climate change. The temperate forests in northeastern China account for more than one-third of both the Chinese forest area and the stocking volume of the national forests, and play a crucial role in the national and global carbon budgets and climatic system (Wang, 2006). The 16 tree species involved in this study coexist in a naturally regenerated forest (45°25′28‴N, 127°38′55″E) nearby the Maoershan forest ecosystem research station of the Northeast Forestry University, Northeast China. However, these species significantly differed in their photosynthetic capacity and water use efficiency (water loss). For instance, *Tilia amurensis*, a simple-leaved species widely distributed at well-drained sites with relatively deep fertile soils, showed higher water use efficiency (95.1 mol H_2_O m^-2^ s^-1^), whereas *Juglans mandshurica*, a compound-leaved species occupying arid and oligotrophic sites, had lower water use efficiency (38.6 mol H_2_O m^-2^ s^-1^; Sang et al., 2011). We, therefore, are very interested in understanding the variation and distribution of leaf, stoma, and twig traits, as well as their relationships with excised leaf water loss of all these species when they co-exist in a temperate forest with the same growth environment. Specifically, we aimed to test the hypothesis that the larger the leaf is, the faster the leaf water loses, because, compared to small leaves, large leaves have more surface area for the loss of water through transpiration.

## Materials and Methods

### Study Site and Species

The present study was conducted in a temperate forest at the Maoershan forest ecosystem research station (45°25′35″N, 127°38′20″E) of the Northeast Forestry University, northeastern China. This study site has a temperate, continental monsoon climate. The mean annual temperate is 2.8°C, with the highest monthly mean temperature of 20.9°C occurring in July and the lowest monthly mean temperature of –19.6 °C occurring in January. The mean annual precipitation is 723 mm, 66% of which falls from June to August. The study site is dominated by the second-growth forest naturally regenerated after the mixed mature *Pinus koraiensis* with broad-leaved trees were harvested over 70 years ago. The soils are classified as Hap-Boric Luvisols, well drained with high organic matter (Gu et al., 2014). For each of the 16 study species in our study, three healthy, adult individual trees were randomly selected in that second-growth forest in September 2013 (Table 1). From each individual tree selected, we collected 3–5 current-year twigs (5–40 cm in length) from the upper sunny part of the tree canopy, giving a total of 9–15 twigs collected for each species. All twigs collected were stored in sealed plastic bags, on ice, in the dark and transported to the laboratory within 1 h for further processing.

**Table 1:** List of sixteen tree species studied in a temperate forest in northeastern China.

No.	Species	Abbreviation	Family	Leaf type
1	*Acer ginnala*	Acgi	Aceraceae	Simple-leaved
2	*Acer mandshuricum*	Acma	Aceraceae	Compound-leaved
3	*Acer mono*	Acmo	Aceraceae	Simple-leaved
4	*Acer tegmentosum*	Acte	Aceraceae	Simple-leaved
5	*Albizia kalkora*	Alka	Leguminosae	Compound-leaved
6	*Betula costata*	Beco	Betulaceae	Simple-leaved
7	*Betula platyphylla*	Tepl	Betulaceae	Simple-leaved
8	*Fraxinus mandschurica*	Frma	Oleaceae	Compound-leaved
9	*Juglans mandshurica*	Juma	Juglandaceae	Compound-leaved
10	*Ostrya japonica*	Osja	Betulaceae	Simple-leaved
11	*Quercus mongolica*	Qumo	Fagaceae	Simple-leaved
12	*Salix pierotii*	Sapi	Salicaceae	Simple-leaved
13	*Syinga reticulata*	Syre	Oleaceae	Simple-leaved
14	*Tilia amurensis*	Tiam	Tiliaceae	Simple-leaved
15	*Ulmus japonica*	Ulja	Ulmaceae	Simple-leaved
16	*Ulmus laciniata*	Ulla	Ulmaceae	Simple-leaved


### Variable Measurements

Leaf size was expressed as average individual leaf projected area and leaf dry mass (measured for the entire leaf for simple-leaved species, and for the leaflet in compound-leaved species). The two parameters on leaf size can be used to estimate various aspects of leaf functioning. For instance, leaf area characterizes leaf energy balance, leaf biomechanical efficiency and mechanical load, while leaf mass estimates leaf construction cost (Niinemets et al., 2007).

For each sample twig, the following parameters were recorded: the number of leaves borne on the twig (LN), the length (TL, mm) and diameter of the middle of twigs (TD, mm). Total projected leaf area borne on the twig (TLA, cm^2^) was measured by scanning all leaves collected from a sample twig using a portable scanner (Canon LiDE 110, Japan) and the pictures were then digitized by using ImageJ software (NIH Image). Leafing intensity (LI, number cm^-3^) was volume-based, calculated as the number of simple leaves (simple-leaved species) or leaflet (compound-leaved species) borne on a twig divided by the twig volume following Kleiman and Aarssen (2007), which could provide a metric comparable among species, representing a measure of relative investment in leaf number. Twig volume was calculated from the length and diameter of the twig by assuming the twig had the dimensions of a cylinder. The leaves were dried to constant mass at 70°C for 48 h and then weighted to acquire total leaf mass (TLM, *g*). Individual leaf area (LA, cm^2^) and individual leaf mass (LM, *g*) were calculated as LA = TLA/LN and LM = TLM/LN, respectively. Specific leaf area (cm^2^ *g*^-1^) was then calculated as leaf area divided by leaf dry mass. The oven-dried leaf samples (70°C for 48 h) were ground to fine powder that was sieved at a 0.5 mm mesh size. Leaf total nitrogen (N), phosphorus (P) and potassium (K) concentration were determined after digesting with H_2_SO_4_-H_2_O_2_, using an elemental analyzer (N and P) and a flame photometer (K).

Three fully expanded leaves per tree were randomly selected to be used for stomatal observation based on the abaxial surface by the nail polish impression method (Franks et al., 2009). The stomatal traits were measured using a Leica DFC 450 camera (Nussloch, Germany) mounted on a Leica DM 2500 microscope at 10–20 × magnification and 20–40 × magnification, respectively. Stomatal length (SL, μm) and stomatal width (SW, μm) were measured as the guard cell length and guard cell pair width based on about forty stomata per tree species, respectively. SL and SW were then used to determine the stomatal size (SS, μm^2^). Stomatal density (SD, number mm^-2^) was calculated as the number of stomata per unit of epidermal surface based on about thirty fields of view per tree species.

From each tree, ten fully expanded leaves were randomly selected to estimate the leaf water loss using the excised leaf method (Mccaig and Romagosa, 1989), with the following details. After sampling, the leaves were stored in icebox and immediately transported to the laboratory and fresh weight of leaves was determined. After weighing, leaves were placed in a dark growth cabinet at 28–30°C with 70% relative humidity, and weighed at 1 h intervals for 6 h. They were then dried at 70°C for 48 h, and weighed to determine the dry mass. Leaf water content (%; the percentage of fresh leaf weight), leaf water loss at every 1 h interval (%; LWL_1_, LWL_2_, LWL_3_, LWL_4_, LWL_5_, LWL_6_), and the total leaf water loss during 6 h (%; LWL_1-6_) were calculated using these weights. The rates of mass loss from all reservoirs can be conveniently expressed by a parameter *k*, which equals the fraction of the stored quantity that is lost per unit time (Jenny et al., 1949; Olson, 1963). The water loss rate from leaves (*k*) was thus estimated using an exponential decay model:

$$X_{t}/X_{o}=e^{-kt}$$

where *X*_t_ is the leaf water content at a given time (t) and *X*_0_ is the initial leaf water content.

### Statistical Analyses

A Shapiro–Wilk test (*shapiro.test* function) was used to test the differences from a normal distribution for leaf size, leafing intensity, stomatal size and density. The skewness and kurtosis were also calculated to describe the distribution shape. Positive and negative values of skewness indicate a distribution is right-skewed and left-skewed, respectively. While kurtosis can measure the extent of which a distribution has a pointy peak or a rounded peak. The kurtosis value of normally distributed data should be around three (Alves-Silva et al., 2018). A linear mixed model was used to determine the variance of twig and leaf traits at both tree species and tree individual level (*lmer* function in *lme4* package). Likewise, linear mixed models were performed to determine the potential relationships of leaf size with leafing intensity, twig diameter, specific leaf area, and leaf water rate (*k*) after log_e_-transformation with tree species as the random factor. There are two values of *R*^2^ which can be calculated according to Nakagawa and Schielzeth (2013), i.e., the marginal *R*^2^ ($R_{m}^{2}$), reflecting the proportion of the variance explained by fixed effects (leafing intensity), and the conditional *R*^2^ ($R_{m}^{2}$), reflecting the variance explained by both fixed and random effects (tree species). Satterthwaite’s approximation and likelihood ratio test were used to estimate the denominator degrees of freedom and *p* values of the fixed effects and the random effects (*lmerTest* package). Multiple-trait relationships were analyzed by principal component analysis (PCA, *princomp* function). Non-parametric tests (*kruskal.test* function) were picked to test the effects of tree species on leaf, twig, and stoma traits, as well as leaf water loss rate (*k*). TukeyC test was chosen to determine differences among tree species when *p* < 0.05. Moreover, Spearman correlation (*cor* and *rcorr* function in *Hmisc* package) was used to determine correlations among all examined traits. All statistical analyses were conducted with R 3.5.1 (R Core Team, 2018).

## Results

**FIGURE 1:**
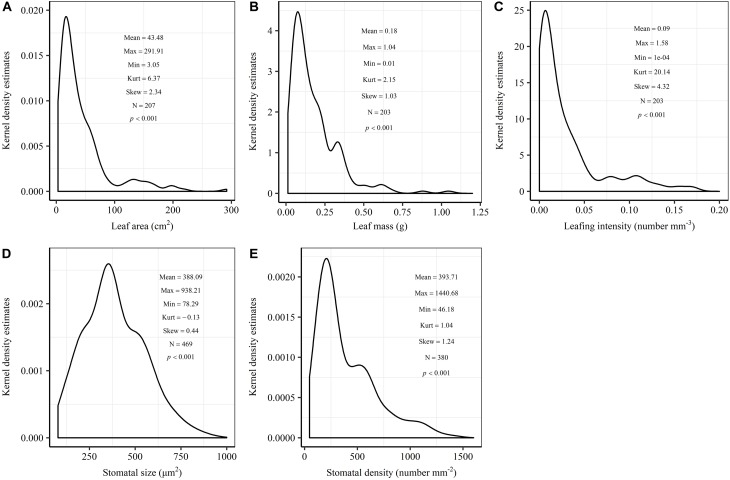
Kernel density estimates of individual leaf area **(A)**, individual leaf mass **(B)**, leafing intensity **(C)**, stomatal size **(D)** and stomatal density **(E)**. The y-axis indicates the abundance of leaf and stomatal traits with a given value. The mean, maximum (Max), minimum (Min), kurtosis (Kurt), skewness (Skew) and sample size (N) were shown in the insets. The frequency distribution was significant different from normal distribution at *p* < 0.05.

**FIGURE 2:**
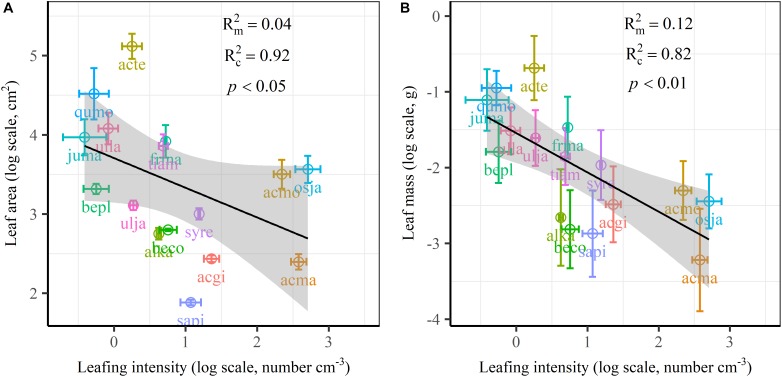
Relationships of volume-based leafing intensity with individual leaf area **(A)** and individual leaf mass **(B)** from linear mixed models with leafing intensity as the mixed factor and tree species as the random factor. Marginal *R*^2^ ($R_{m}^{2}$) reflects the proportion of the variance explained by fixed factors and conditional R^2^ ($R_{c}^{2}$) reflects the proportion of the variance explained by both fixed and random factors. Average value per tree species and its 0.1-fold standard error were given. Gray bands show 95% confidence intervals. The abbreviations of tree species were provided in Table 1.

The frequency distribution from Shapiro–Wilk test yielded high right-asymmetry for leaf size (leaf area and leaf mass; Figure 1A,B), leafing intensity (Figure 1C) as well as stomatal size and stomatal density (Figure 1D,E) when all the species sampled were pooled. The positive skewness indicated that the distributions were right-skewed (Figure 1). Furthermore, the distribution patterns for leaf area and leafing intensity (kurtosis > 3) were leptokurtic with a narrow peak (Figure 1A,C). Average individual leaf area and individual leaf mass across species varied by two and one orders of magnitude, ranging from 6.58 ± 0.71 cm^2^ (*Salix pierotii*) to 166.83 ± 4.90 cm^2^ (*Acer tegmentosum*), and from 0.04 ± 0.01 *g* (*Acer mandshuricum*) to 0.50 ± 0.01 *g* (*Acer tegmentosum*), respectively (Table 2). Leafing intensity was significantly negatively correlated with leaf size, accounting for 4% of variation in individual leaf area (marginal *R*^2^; $R_{m}^{2}$ 0.04, *p* < 0.05) and 12% of variation in individual leaf mass ($R_{m}^{2}$ = 0.12, *p* < 0.01) (Figure 2). The majority of the variance was explained by the random effects (tree species), as indicated by the large difference between $R_{m}^{2}$ and $R_{c}^{2}$ (conditional *R*^2^; $R_{c}^{2}$ = 0.92 for leaf area, $R_{c}^{2}$ = 0.82 for leaf mass, Figure 2). Across all species, there were marginally and significantly positive relationships between individual leaf area ($R_{m}^{2}$ = 0.06, $R_{c}^{2}$ = 0.93, *p* = 0.09), individual leaf mass ($R_{m}^{2}$ = 0.20, $R_{c}^{2}$ = 0.87, *p* < 0.01) and the corresponding twig diameter, respectively. Specific leaf area varied approximately three-fold, ranging from 123.79 ± 17.44 cm^2^ *g*^-1^ (*Salix pierotii*) to 370.15 ± 22.31 cm^2^ *g*^-1^ (*Ostrya japonica*; Table 2), and specific leaf area was positively correlated with individual leaf area ($R_{m}^{2}$ = 0.16, $R_{c}^{2}$ = 0.80, *p* < 0.05) but not related with individual leaf mass (*p* > 0.05). Besides, the variance of twig, leaf and stoma traits in our study was strongly dependent on species identity (Supplementary Table S1).

**Table 2:** Descriptive statistics (mean ± 1SE, *n* = 3) for twig, leaf and stoma traits for the 16 tree species studied.

Species	TL	TD	LI	TLA	LA	TLM	LM	SLA	*N*	*P*	*K*	*SD*	*SS*
Acgi	162.88 ± 27.01^ab^	2.58 ± 0.22^d^	3.90 ± 0.34^b^	154.15 ± 35.65^b^	11.42 ± 1.53^de^	1.21 ± 0.22^c^	0.08 ± 0.01^d^	141.37 ± 10.09^f^	No data				
Acma	46.16 ± 7.98^b^	1.90 ± 0.07^d^	13.18 ± 2.92^a^	176.95 ± 10.26^b^	10.98 ± 0.38^de^	0.63 ± 0.05^c^	0.04 ± 0.01^d^	289.41 ± 9.57^abc^	2.56 ± 0.12^abcd^	0.27 ± 0.02^b^	0.98 ± 0.04^d^	401.14 ± 61.92^cd^	656.46 ± 16.62^ab^
Acmo	26.85 ± 8.61^b^	1.80 ± 0.07^d^	10.42 ± 3.13^ab^	196.22 ± 40.90^b^	33.17 ± 6.26^cde^	0.55 ± 0.11^c^	0.10 ± 0.02^d^	362.67 ± 8.37^a^	2.15 ± 0.23^d^	0.26 ± 0.05^b^	1.18 ± 0.13^cd^	195.05 ± 43.29^efg^	379.40 ± 19.19^cdef^
Acte	25.42 ± 5.00^b^	2.91 ± 0.05^d^	1.29 ± 0.25^b^	333.65 ± 9.81^b^	166.83 ± 4.90^a^	1.01 ± 0.03^c^	0.50 ± 0.01^a^	337.03 ± 2.44^ab^	2.41 ± 0.08^bcd^	0.35 ± 0.01^ab^	1.42 ± 0.09^abcd^	207.49 ± 15.62^efg^	438.06 ± 33.41^cd^
Alka	278.06 ± 71.55^a^	4.78 ± 0.65^c^	1.87 ± 0.74^b^	1187.90 ± 233.02^b^	15.67 ± 2.11^de^	5.43 ± 1.07^c^	0.07 ± 0.01^d^	219.68 ± 10.29^cdef^	3.17 ± 0.08^a^	0.39 ± 0.02^ab^	1.78 ± 0.10^abc^	331.00 ± 83.19^de^	416.34 ± 36.88^cde^
Beco	164.92 ± 16.57^ab^	1.69 ± 0.09^d^	2.13 ± 0.30^b^	131.30 ± 16.18^b^	16.44 ± 1.18^cde^	0.48 ± 0.07^c^	0.06 ± 0.01^d^	295.32 ± 6.08^abc^	2.66 ± 0.01^abcd^	0.35 ± 0.01^ab^	1.74 ± 0.05^abc^	293.47 ± 18.70^def^	251.38 ± 19.37^fg^
Bepl	186.75 ± 21.32^ab^	2.57 ± 0.24^d^	0.78 ± 0.17^b^	194.94 ± 27.57^b^	27.56 ± 1.89^cde^	1.15 ± 0.20^c^	0.17 ± 0.02^cd^	173.12 ± 8.19^ef^	2.91 ± 0.08^ab^	0.34 ± 0.04^ab^	1.16 ± 0.07^cd^	264.44 ± 13.80^def^	475.89 ± 9.98^cd^
Frma	195.83 ± 92.19^ab^	7.01 ± 1.01^b^	2.06 ± 0.87^b^	4752.40 ± 558.04^a^	50.28 ± 7.76^bcd^	21.24 ± 2.49^b^	0.23 ± 0.01^bcd^	242.86 ± 16.08^bcde^	2.93 ± 0.20^ab^	0.37 ± 0.07^ab^	1.34 ± 0.02^bcd^	571.81 ± 23.32^ab^	274.78 ± 5.44^efg^
Juma	158.33 ± 18.33^ab^	10.69 ± 0.19^a^	0.66 ± 0.05^b^	5975.82 ± 802.73^a^	52.90 ± 9.71^bcd^	31.08 ± 4.17^a^	0.33 ± 0.01^ab^	201.66 ± 3.86^cdef^	2.84 ± 0.07^abc^	0.37 ± 0.02^ab^	2.03 ± 0.13^ab^	295.57 ± 28.96^def^	373.43 ± 8.64^cdefg^
Osja	25.49 ± 15.14^b^	1.62 ± 0.05^d^	14.98 ± 5.78^a^	116.85 ± 0.45^b^	35.31 ± 5.51^cde^	0.32 ± 0.02^c^	0.09 ± 0.03^d^	370.15 ± 22.31^a^	2.09 ± 0.21^d^	0.09 ± 0.00^c^	1.62 ± 0.27^abcd^	98.63 ± 10.75^g^	412.85 ± 11.65^cde^
Qumo	118.58 ± 4.88^ab^	2.94 ± 0.19^cd^	0.76 ± 0.13^b^	519.82 ± 132.90^b^	91.67 ± 25.46^b^	2.19 ± 0.31^c^	0.39 ± 0.10^ab^	233.84 ± 6.46^bcde^	2.56 ± 0.02^abcd^	0.35 ± 0.02^ab^	1.36 ± 0.08^abcd^	518.29 ± 23.69^bc^	348.86 ± 14.14^defg^
Sapi	161.12 ± 6.84^ab^	2.09 ± 0.09^d^	2.92 ± 0.24^b^	114.34 ± 4.64^b^	6.58 ± 0.71^e^	0.91 ± 0.06^c^	0.06 ± 0.00^d^	123.79 ± 17.44^f^	2.56 ± 0.10^abcd^	0.44 ± 0.02^a^	1.80 ± 0.09^abc^	217.39 ± 27.74^efg^	224.56 ± 21.77^g^
Syre	118.50 ± 10.48^ab^	2.29 ± 0.06^d^	3.28 ± 0.88^b^	137.43 ± 15.39^b^	20.12 ± 2.06^cde^	0.96 ± 0.08^c^	0.14 ± 0.01^cd^	148.41 ± 7.47^def^	No data				
Tiam	65.63 ± 17.07^b^	2.14 ± 0.17^d^	1.99 ± 0.53^b^	188.53 ± 23.72^b^	47.42 ± 4.37^cde^	0.63 ± 0.11^c^	0.16 ± 0.02^cd^	302.35 ± 14.57^abc^	2.75 ± 0.20^abcd^	0.27 ± 0.02^b^	1.66 ± 0.32^abcd^	149.08 ± 10.63^fg^	416.64 ± 35.95^cde^
Ulja	174.70 ± 43.87^ab^	2.65 ± 0.38^d^	1.31 ± 0.62^b^	227.34 ± 53.46^b^	22.42 ± 1.83^cde^	1.93 ± 0.49^c^	0.20 ± 0.03^cd^	184.84 ± 58.30^def^	2.25 ± 0.06^cd^	0.32 ± 0.02^ab^	1.26 ± 0.06^cd^	697.00 ± 37.82^a^	516.43 ± 20.20^bc^
Ulla	134.08 ± 46.13^ab^	2.60 ± 0.23^d^	0.92 ± 0.25^b^	311.38 ± 42.08^b^	59.11 ± 7.44^bc^	1.12 ± 0.13^c^	0.22 ± 0.05^bcd^	286.88 ± 22.14^abcd^	2.54 ± 0.04^abcd^	0.36 ± 0.03^ab^	2.07 ± 0.08^a^	219.17 ± 9.89^efg^	688.92 ± 45.73^a^


*TL, the length of twig (mm); TD, the diameter of twig (mm); LI, leafing intensity (100^∗^number cm^-*3*^); TLA, total leaf area per twig (cm^*2*^); LA, individual leaf area (cm^*2*^); TLM, total leaf mass per twig (g); LM, individual leaf mass (g); SLA, specific leaf area (cm^*2*^ g^-*1*^); N, leaf nitrogen concentration (%); P, leaf phosphorus concentration (%); K, leaf potassium concentration (%); SD, stomatal density (number mm^-*2*^); SS, stomatal size (μm^*2*^). The abbreviations of tree species were shown in Table 1. Different lower case letters indicate significant differences of twig, leaf and stoma traits among tree species at *p* < 0.05.*

**FIGURE 3:**
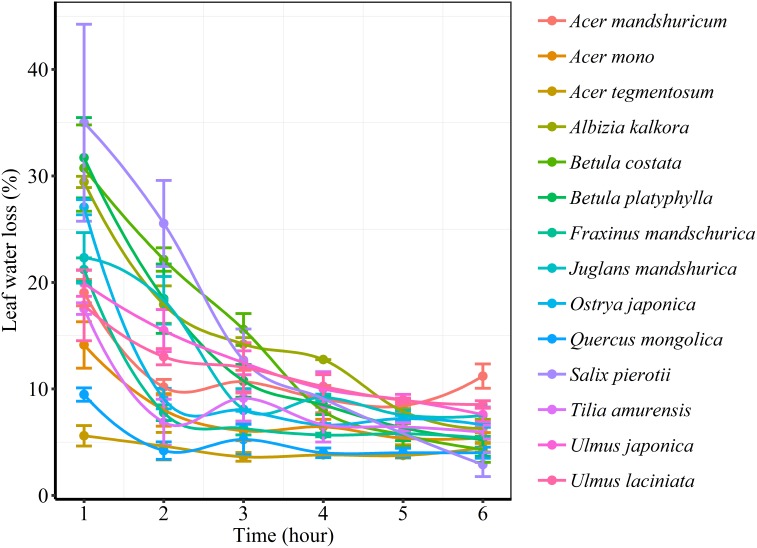
Changes in leaf water loss (%) of different tree species over time.

**FIGURE 4:**
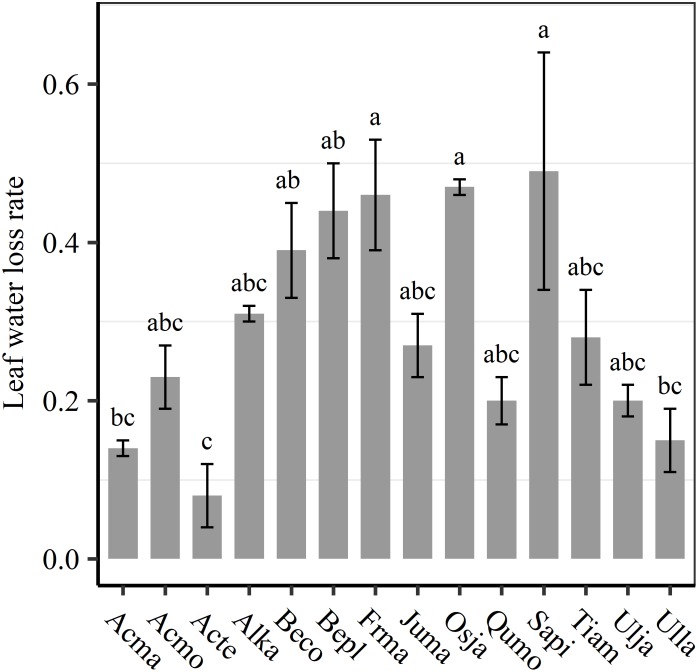
Differences in leaf water loss rate (*k*) among tree species. Different lower case letters indicate significant differences in leaf water loss rate (*k*) among tree species at *p* < 0.05. The abbreviations of tree species were shown in Table 1.

**FIGURE 5:**
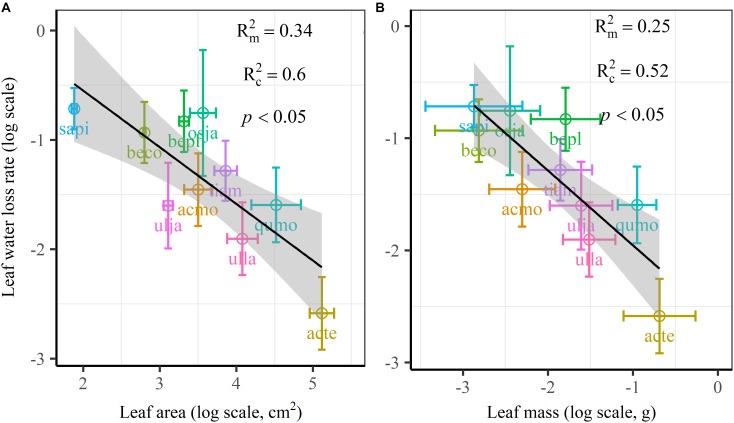
Leaf water loss rate (*k*) in relation to individual leaf area **(A)** and individual leaf mass **(B)** of 10 simple-leaved tree species, analyzed using linear mixed models with individual leaf area or mass as the mixed factor and tree species as the random factor. Marginal *R*^2^ ($R_{m}^{2}$) reflects the proportion of the variance explained by fixed factors and conditional *R*^2^ ($R_{c}^{2}$) reflects the proportion of the variance explained by both fixed and random factors. Average value per tree species and its 0.1-fold standard error were given. Gray bands show 95% confidence intervals. The abbreviations of tree species were shown in Table 1.

**FIGURE 6:**
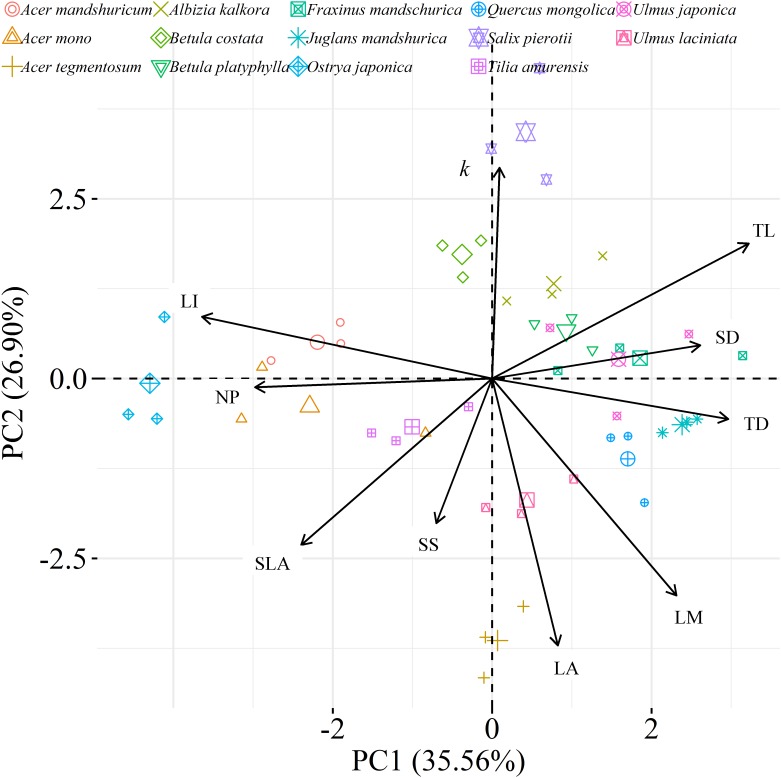
Principal component analysis (PCA) among leaf, twig, stoma traits and leaf water loss rate as well as species distribution in the two-dimensional trait space [three points of tree species values (smaller points) and their average values (larger points)]. All data were log_e_-transformed before analysis. *k*, leaf water loss rate; TL, the length of twig; TD, the diameter of twig; LI, leafing intensity; SLA, specific leaf area; SS, stomatal size; SD, stomatal density; LA, individual leaf area; LM, individual leaf mass; NP, the ratio of leaf nitrogen to phosphorus concentration.

We found significant decreases in leaf water loss over time for all tree species (Figure 3). Significant differences in leaf water loss rate (*k*) were detected among tree species (Figure 4). LWL_1_ was the highest in *Salix pierotii* (35%), which lost 90% of leaf water content during 6 h (LWL_1-6_), resulting in the highest leaf water loss rate (*k*, Figure 3, 4). However, LWL_1_ and LWL_1-6_ of *Acer tegmentosum* were only 5.6% and 26% of leaf water content, therefore, the lowest value in leaf water loss rate (*k*, Figure 3, 4). Leaf water loss was strongly negatively correlated with individual leaf area and individual leaf mass (with an exception for LWL_2_) from 1 (LWL_1_) to 4 h (LWL_4_; Table 3). The 38 and 30% of variation in LWL_1-6_ were accounted by individual leaf area and individual leaf mass, respectively (Table 3). Leaf water loss rate (*k*) significantly linearly decreased with increasing individual leaf area and individual leaf mass for the simple-leaved tree species (Figure 5), but *k* was not related to initial leaf water content when all species pooled together (Supplementary Table S2). Furthermore, during the first 4 h of measurement (LWL_1_ to LWL_4_), leaf water loss was correlated with neither stomatal size nor stomatal density (Supplementary Table S2). LWL_5_ and LWL_6_ were markedly positively correlated with stomatal size, but leaf water loss rate (*k*) was significantly negatively related with stomatal size (Supplementary Table S2). The first axis of principal component analysis accounted for 35.6% of total variation, showing strong loadings on twig length, twig diameter, ratio of leaf nitrogen to phosphorus concentration, stomatal density and leafing intensity (Figure 6 and Supplementary Table S3). The second axis, which accounted for 26.9% of the total variation, had strong loading on individual leaf area, specific leaf area, individual leaf mass and leaf water loss rate (Figure 6 and Supplementary Table S3). Leaf water loss rate (*k*), stomatal density, and stomatal size had high scores on the third axis (Supplementary Table S3).

**Table 3:** Relationships between leaf size (individual leaf area, LA and individual leaf mass, LM) and leaf water loss from 1 h (LWL_1_) to 6 h (LWL_6_; *n* = 3), analyzed using linear mixed models with LA and LM as the mixed factor and tree species as the random factor.

	LA						LM					
		
	Slope	*p*	Intercept	*p*	$R_{m}^{2}$	$R_{c}^{2}$	Slope	*p*	Intercept	*p*	$R_{m}^{2}$	$R_{c}^{2}$
LWL_1_	**–0.34**	**0.002**	**4.12**	**< 0.001**	**0.34**	**0.76**	**–0.23**	**0.034**	**2.51**	**< 0.001**	**0.13**	**0.77**
LWL_2_	**–0.38**	**0.005**	**3.72**	**< 0.001**	**0.30**	**0.72**		0.102		< 0.001		
LWL_3_	**–0.37**	**< 0.001**	**3.44**	**< 0.001**	**0.43**	**0.62**	**–0.32**	**< 0.001**	**1.53**	**< 0.001**	**0.28**	**0.62**
LWL_4_	**–0.23**	**0.011**	**2.79**	**< 0.001**	**0.25**	**0.56**	**–0.21**	**0.026**	**1.57**	**< 0.001**	**0.17**	**0.61**
LWL_5_		0.056		< 0.001				0.098		< 0.001		
LWL_6_		0.71		0.002				0.679		< 0.001		
LWL_1-6_	**–0.25**	**< 0.001**	**4.90**	**< 0.001**	**0.38**	**0.85**	**–0.17**	**0.012**	**3.72**	**< 0.001**	**0.30**	**0.56**


*Marginal *R*^*2*^ ($R_{m}^{2}$) reflects the proportion of the variance explained by fixed factors, and conditional R^*2*^ ($R_{c}^{2}$) reflects the proportion of the variance explained by both fixed and random factors. Significant relationships were highlighted in bold.*

## Discussion

In this study, we observed that the distributions of leaf size, leafing intensity, stomatal size and stomatal density at community-level were noticeably skewed to the right, with a long tail of larger values (Figure 1). Right-skewed distributions in plant assemblages have been reported recently for plant height (Moles et al., 2009), seed size (Moles et al., 2006), leaf size (Milla, 2009) and fine root diameter (Ma et al., 2018) at various scales (Ogawa, 2008; Dombroskie and Aarssen, 2010; Whitman and Aarssen, 2010). The right-skewed distributions at different taxonomical levels reported for natural vegetations indicate a pervasive signal for adaptive size metrics (Dombroskie and Aarssen, 2010). Thus, it is greatly necessary to explicitly examine the frequency distribution in the extremely similar fashion. The preponderance of small leaves (namely the right-skewed unimodal leaf size distribution) is considered to be a consequence of the left-wall effect, because the sizes of things must be greater than zero (Aarssen et al., 2006; Jensen and Zwieniecki, 2013). Most habitats for terrestrial plants have environmental conditions where adaptation is conferred through physiological optimization associated directly with relatively small leaf size (Kleiman and Aarssen, 2007). We found that the distribution for leaf area (kurtosis = 6.32) was leptokurtic with a narrow peak in our temperate forest in northeastern China (Figure 1A). However, platykurtic distributions of leaf area (lower kurtosis value) are observed and their degrees of platykurtosis decreased with decreased soil water in woody plant communities at Jasper Ridge, California (Cornwell and Ackerly, 2009). Skewness and kurtosis of community trait distribution are highly sensitive to climate, soil and topography (Le Bagousse-Pinguet et al., 2016). Such leptokurtosis of all examined traits in our study potentially arise from the environmental heterogeneity of habitats (Sang et al., 2011) or the existence of variations on leaf traits among the 16 tree species (Supplementary Table S1).

There are several well-known compromises between allocation to size and number of organs in plant bodies, or of individuals in plant populations (Yang et al., 2008; Whitman and Aarssen, 2010; Scott and Aarssen, 2012). The results in this study reinforced overall generality of a cross-species trade-off between the number of leaves attached to a unit of yearly twig and the size of individual leaves in woody species. Leafing intensity is a whole-plant morphological trait, which can provide remarkable explanatory power in accounting for a fundamental pattern of leaf functional trait variation (Whitman and Aarssen, 2010). Therefore, a “leafing intensity premium” hypothesis has been proposed with supporting evidence of the right-skewed distribution of leaf size frequency (Kleiman and Aarssen, 2007). However, if high leafing intensity confers important general fitness advantages, why then do most species not have relatively high leafing intensity (namely the left-skewed frequency distribution)? Actually the frequency distribution of leafing intensity is also right-skewed (Figure 1C), which violates the assumption of leafing intensity premium that leafing intensity is left-skewed. Based on dataset covering 224 species, a similar right-skewed distribution of leafing intensity was also observed (Milla, 2009). In fact, both leaf size and leafing intensity may be direct products of natural optimizing selection (Kleiman and Aarssen, 2007; Tozer et al., 2015). Decreasing the cost of the associated twigs by deploying a given leaf mass as fewer, larger leaves, considered as the selective advantage of lower leafing intensity (Wright et al., 2017). Variation in leaf size and leaf number of plants is determined by a very precise and inevitable resource allocation trade-off relationship (Aarssen, 2012). Moreover, conditional *R*^2^ values were quite high, whereas marginal *R*^2^ values were quite low for the associations of leafing intensity with individual leaf area and individual leaf mass (Figure 2 and Supplementary Figure S1). This indicated that the leafing intensity could not solely account for the large variation in leaf size. Given the relatively small sample with only 16 species in our study, it is very necessary to enlarge the scope of plant families to claim wide generality of leaf size-number trade-off relationship, evaluating comprehensively the adaptive significance of the “leafing intensity premium” hypothesis.

Our results showed that the size of individual leaves across species was correlated with twig size (determined as the diameter of twigs; Supplementary Table S2). Thus the leaf size-number trade-off is also linked to Corner’s Rules (Corner, 1949). As predicted by Corner’s Rules, thin twigs bear scarcely separated nodes, with many small leaves per twig unit and vice versa for thick twigs (Corner, 1949; Pickup et al., 2005). The twig size of different tree species may influence leafing intensity and leaf size with an endogenous mechanism (Yang et al., 2008). Hence, there exists a leaf size-twig size spectrum (LSTSS), which extends from species with small leaves, small twigs and close ramification to species with large leaves, thick twigs and less frequent branching (Pickup et al., 2005; Dias et al., 2017). However, because of strong correlation among leaf size, leafing intensity and twig size across species, small leaves may be attributed to natural selection favoring either small leaves, high leafing intensity or small twigs. It is difficult to distinguish the mechanism controlling leaf size variation and/or how these mechanisms interactively influence leaf size evolution (Yang et al., 2008). But the current-year twigs have the property of permitting the leaf size-number trade-off relationship to be detected, because they include only the annual growth of the plant species with very low levels of secondary growth (Yang et al., 2008). Consequently, it is very necessary to propose synthetic approaches involving multiple scales such as leaf, twig, even whole-tree scaling, to thoroughly comprehend leaf size variation.

The most striking and potentially important pattern found in this work was the strongly negative relationship between leaf size and leaf water loss for all tree species (Table 3) as well as leaf water loss rate (*k*) for the simple-leaved tree species (Figure 5 and Supplementary Figure S2), these results rejected our initial hypothesis that the larger the leaf is, the faster the leaf water loses. This finding furthermore highlighted a fundamental difference in leaf thermal regulation between small and large. Smaller exhibited the faster leaf water loss, which was effective in shedding heat, obtaining an adaptive advantage to high light intensity or hot environments, where smaller leaves were dominated (Leigh et al., 2017). Larger leaf species might incur higher costs in water-sourcing root biomass to supply the transpiration needed to cool leaves (Givnish and Vermeij, 1976; Yates et al., 2010). Smaller leaves intercepting more solar radiation in the upper part of the canopy have higher rates of carbon assimilation, water loss and thus are physiologically more active (Boardman, 1977). Previous studies have confirmed that the variation in leaf size can substantially modify the whole-leaf integrated photosynthetic activity, namely overall higher mass-based photosynthetic activity of smaller leaves (Poorter and Evans, 1998; Niinemets et al., 2006, 2007). Thus small leaves must ensure greater leaf hydraulic conductance to maintain greater photosynthesis (Scoffoni et al., 2011). This may be a particularly important strategy for driving nutrient mass-flow from the roots of plants that take up most of their nutrients (Cramer et al., 2009; Yates et al., 2010). However, large leaf may have fitness benefits derived from a greater boundary layer thickness for heat exchange, allowing leaves to more quickly heat up to favorable temperatures for photosynthesis, thus maximizing photosynthetic returns under cooler environments, such as cool mornings (Michaletz et al., 2016; Wright et al., 2017). Restriction of leaf water loss through the plant cuticle for large leaf species during periods of severe water stress is an important drought survival mechanism (Clarke et al., 1991). However, it may be noteworthy to mention that, due to the limited species number, we could not analyze the relationships between leaf water loss rate (*k*) and leaf size for the four compound-leaved tree species in our study. Further studies with more compound-leaved tree species are needed to better identify the leaf size-leaf water loss relationships at both leaflet and single leaf level.

Leaf water loss occurs as stomatal and cuticular transpiration (Hall and Jones, 1961; Basal et al., 2005). The initial period of leaf water loss is assumed to be due to stomatal transpiration, and the later water loss (after stomatal closure) presumably is due to cuticular transpiration (Hall and Jones, 1961). Therefore, stomatal behavior is critical for regulating water fluxes of plants in terrestrial ecosystems (Reich and Borchert, 1988; Basal et al., 2005; Anderegg et al., 2018). In our study, the relationships between leaf water loss and stomatal size varied from non-significant relationship (LWL_1_ to LWL_4_) to significantly positive one (LWL_5_ and LWL_6_; Supplementary Table S2). Additionally, leaf water loss seemed to be not correlated with stomatal density. The contrasting associations indicated that stomatal characteristics in our study might be not important or sophisticated factors influencing observed leaf size-related differences in leaf water loss. This was proved by the important loadings of stomatal size and stomatal density on the third axis of principal component analysis (Supplementary Table S3). Moreover, residual stomatal transpiration after complete stomatal closure had been identified as the major determinant of cuticular transpiration for some species (Burghardt and Riederer, 2003). In our study, however, we failed to make critical distinctions between stomatal and cuticular transpiration. Certainly, the loss of leaf water might be related to epicuticular wax, glaucousness or leaf rolling, which are not studied in this study (Hall and Jones, 1961; Cameron et al., 2006). In conclusion, based on our findings of increasing leaf water loss with decreasing leaf size, it was speculated that the small leaf probably exhibited the advantage in leaf temperature regulation. So we agree with the theory that leaves of small size have adaptive value for plants evolved for hot environments. This knowledge has the potential to enrich vegetation models, in which leaf temperature and water balance during photosynthesis play key roles in, potentially, contributing to well-known biogeographic trends in leaf size.

## Author Contributions

CW conceived the ideas. JH, YC, and GW collected the data. CW, and T-HZ performed the analysis. CW, BS, and XY wrote the first draft. WG and M-HL led the writing of the manuscript. This work has been approved for publication by all co-authors.

## Conflict of Interest Statement

The authors declare that the research was conducted in the absence of any commercial or financial relationships that could be construed as a potential conflict of interest.
