# Supplementary material for: The Smaller the Leaf Is, the Faster the Leaf Water Loses in a Temperate Forest
Source: Front Plant Sci. 2019 Feb 4;10:58. doi: 10.3389/fpls.2019.00058 (PMC6369174; doi:10.3389/fpls.2019.00058)
Supplement: Supplementary file 1 [file Table_1.docx]

Supplementary information

Table S1 Proportion of variance (%) explained at tree species and tree individual level for twig, leaf and stoma traits

|  | No. | Species | Individual | Residual |
| --- | --- | --- | --- | --- |
| The length of twig (TL) | 204 | 45.83 | 15.63 | 38.54 |
| The diameter of twig (TD) | 204 | 62.03 | 10.13 | 27.85 |
| Leafing intensity (LI) | 204 | 47.42 | 12.68 | 39.91 |
| Total leaf area per twig (TLA) | 204 | 65.96 | 6.38 | 27.66 |
| Individual leaf area (LA) | 208 | 60.28 | 13.48 | 26.24 |
| Total leaf mass per twig (TLM) | 204 | 69.19 | 0.00 | 30.81 |
| Individual leaf mass (LM) | 204 | 57.81 | 13.28 | 28.91 |
| Specific leaf area (SLA) | 208 | 54.55 | 12.12 | 33.33 |
| Stomatal density (SD) | 381 | 67.33 | 10.89 | 21.78 |
| Stomatal size (SS) | 471 | 58.97 | 8.97 | 32.05 |

Table S2 Coefficients of Spearman’s correlation for all examined traits.

|  | TL | TD | LN | LI | TA | LA | LM | TM | SLM | SLA | NP | N | P |
| --- | --- | --- | --- | --- | --- | --- | --- | --- | --- | --- | --- | --- | --- |
| TL | 1.00 |  |  |  |  |  |  |  |  |  |  |  |  |
| TD | 0.51^***^ | 1.00 |  |  |  |  |  |  |  |  |  |  |  |
| LN | 0.64^***^ | 0.46^**^ | 1.00 |  |  |  |  |  |  |  |  |  |  |
| LI | -0.64^***^ | -0.73^***^ | -0.12 | 1.00 |  |  |  |  |  |  |  |  |  |
| TA | 0.30 | 0.91^***^ | 0.34^*^ | -0.61^***^ | 1.00 |  |  |  |  |  |  |  |  |
| LA | -0.25 | 0.42^**^ | -0.46^**^ | -0.48^**^ | 0.56^***^ | 1.00 |  |  |  |  |  |  |  |
| LM | 0.04 | 0.65^***^ | -0.23 | -0.70^***^ | 0.69^***^ | 0.87^***^ | 1.00 |  |  |  |  |  |  |
| TM | 0.58^***^ | 0.95^***^ | 0.53^***^ | -0.69^***^ | 0.86^***^ | 0.32^*^ | 0.63^***^ | 1.00 |  |  |  |  |  |
| SLM | 0.71^***^ | 0.46^**^ | 0.56^***^ | -0.43^**^ | 0.16 | -0.31^*^ | 0.11 | 0.60^***^ | 1.00 |  |  |  |  |
| SLA | -0.65^***^ | -0.46^**^ | -0.55^***^ | 0.40^**^ | -0.18 | 0.28 | -0.09 | -0.59^***^ | -0.95^***^ | 1.00 |  |  |  |
| NP | -0.39^*^ | -0.25 | -0.06 | 0.41^**^ | -0.18 | -0.10 | -0.29 | -0.37^*^ | -0.39^*^ | 0.33^*^ | 1.00 |  |  |
| N | 0.54^***^ | 0.50^***^ | 0.53^***^ | -0.34^*^ | 0.41^**^ | -0.08 | 0.02 | 0.47^**^ | 0.39^*^ | -0.48^**^ | 0.01 | 1.00 |  |
| P | 0.60^***^ | 0.45^**^ | 0.35^*^ | -0.48^**^ | 0.33^*^ | 0.00 | 0.19 | 0.53^***^ | 0.50^***^ | -0.50^***^ | -0.82^***^ | 0.46^**^ | 1.00 |
| K | 0.24 | 0.14 | 0.11 | -0.19 | 0.08 | 0.06 | 0.04 | 0.15 | 0.04 | -0.10 | -0.16 | 0.17 | 0.34^*^ |
| LWL_1_ | 0.54^***^ | -0.07 | 0.51^***^ | 0.07 | -0.26 | -0.58^***^ | -0.50^***^ | -0.02 | 0.39^*^ | -0.41^**^ | 0.10 | 0.42^**^ | 0.19 |
| LWL_2_ | 0.64^***^ | 0.02 | 0.51^***^ | -0.10 | -0.20 | -0.60^***^ | -0.38^*^ | 0.11 | 0.55^***^ | -0.54^***^ | -0.24 | 0.26 | 0.40^**^ |
| LWL_3_ | 0.47^**^ | -0.18 | 0.30 | 0.04 | -0.30 | -0.62^***^ | -0.52^***^ | -0.10 | 0.35^*^ | -0.32^*^ | -0.11 | 0.13 | 0.22 |
| LWL_4_ | 0.41^**^ | 0.06 | 0.44^**^ | 0.00 | -0.04 | -0.49^**^ | -0.37^*^ | 0.14 | 0.37^*^ | -0.39^*^ | 0.01 | 0.18 | 0.13 |
| LWL_5_ | 0.22 | 0.04 | 0.30 | -0.02 | 0.02 | -0.32^*^ | -0.25 | 0.08 | 0.19 | -0.13 | 0.15 | 0.03 | -0.11 |
| LWL_6_ | -0.05 | 0.03 | 0.17 | 0.05 | 0.11 | -0.12 | -0.12 | 0.02 | -0.05 | 0.09 | 0.33^*^ | -0.09 | -0.35^*^ |
| TWL | 0.49^***^ | 0.03 | 0.57^***^ | 0.08 | -0.22 | -0.71^***^ | -0.50^***^ | 0.10 | 0.58^***^ | -0.58^***^ | -0.01 | 0.25 | 0.20 |
| LWC | -0.26 | 0.12 | -0.14 | 0.16 | 0.24 | 0.35^*^ | 0.11 | 0.00 | -0.52^***^ | 0.47^**^ | 0.21 | -0.02 | -0.15 |
| *k* | 0.36^*^ | -0.06 | 0.32^*^ | 0.04 | -0.23 | -0.26 | -0.26 | -0.04 | 0.24 | -0.27 | 0.15 | 0.36^*^ | 0.09 |
| SS | -0.19 | -0.02 | -0.26 | -0.06 | 0.04 | 0.09 | 0.04 | -0.09 | -0.17 | 0.21 | 0.22 | -0.26 | -0.34^*^ |
| SD | 0.41^**^ | 0.41^**^ | 0.52^***^ | -0.27 | 0.40^**^ | -0.12 | 0.16 | 0.54^***^ | 0.52^***^ | -0.45^**^ | -0.21 | 0.15 | 0.19 |

Continued to Table S2.

|  | K | LW1 | LW2 | LW3 | LW4 | LW5 | LW6 | TWL | LWC | *k* | SS | SD |
| --- | --- | --- | --- | --- | --- | --- | --- | --- | --- | --- | --- | --- |
| K | 1.00 |  |  |  |  |  |  |  |  |  |  |  |
| LWL_1_ | 0.20 | 1.00 |  |  |  |  |  |  |  |  |  |  |
| LWL_2_ | 0.36^*^ | 0.73^***^ | 1.00 |  |  |  |  |  |  |  |  |  |
| LWL_3_ | 0.27 | 0.61^***^ | 0.72^***^ | 1.00 |  |  |  |  |  |  |  |  |
| LWL_4_ | 0.33^*^ | 0.45^**^ | 0.67^***^ | 0.70^***^ | 1.00 |  |  |  |  |  |  |  |
| LWL_5_ | 0.19 | 0.25 | 0.34^*^ | 0.46^**^ | 0.73^***^ | 1.00 |  |  |  |  |  |  |
| LWL_6_ | 0.02 | -0.03 | 0.04 | 0.20 | 0.53^**^ | 0.89^***^ | 1.00 |  |  |  |  |  |
| TWL | 0.14 | 0.78^***^ | 0.78^***^ | 0.66^***^ | 0.71^***^ | 0.49^**^ | 0.22 | 1.00 |  |  |  |  |
| LWC | 0.22 | -0.12 | -0.38^*^ | -0.21 | -0.20 | -0.13 | -0.07 | -0.36^*^ | 1.00 |  |  |  |
| *k* | 0.03 | 0.76^***^ | 0.37^*^ | 0.15 | -0.05 | -0.20 | -0.39^*^ | 0.38^*^ | 0.02 | 1.00 |  |  |
| SS | -0.22 | -0.20 | -0.11 | 0.11 | 0.30 | 0.60^***^ | 0.72^***^ | -0.08 | 0.02 | -0.50^***^ | 1.00 |  |
| SD | -0.30 | -0.01 | 0.14 | 0.14 | 0.16 | 0.15 | 0.15 | -0.10 | 0.27 | -0.13 | -0.06 | 1.00 |

TL, the length of twig; TD, the diameter of twig; LN, the number of leaves per twig; LI, leafing intensity; TA, total leaf area per twig; LA, individual leaf area; TM, total leaf mass per twig; LM, individual leaf mass; SLM, specific leaf mass; SLA, specific leaf area; NP, the ratio of leaf nitrogen to phosphorus concentration; N, leaf nitrogen concentration; P, leaf phosphorus concentration; K, leaf potassium concentration; LWL_i_, leaf water loss at i hour; TWL, total leaf water loss for 6 hour; LWC, initial leaf water content; *k*, leaf water loss rate; SD, stomatal density; SS, stomatal size. Correlations were significant at: ^***^*p* < 0.001; ^**^ *p* < 0.01; ^*^*p* < 0.05.

Table S3 The proportion of variation explained (top table) and loading scores of variables on each component (bottom table) from principal component analysis (PCA). The abbreviations of variables were provided in Table S3.

| Components | Eigenvalue | Proportion (%) | Cumulative (%) |
| --- | --- | --- | --- |
| 1 | 3.56 | 35.56 | 35.56 |
| 2 | 2.69 | 26.90 | 62.46 |
| 3 | 1.24 | 12.44 | 74.90 |
| 4 | 0.82 | 8.20 | 83.10 |
| 5 | 0.61 | 6.12 | 89.22 |
| 6 | 0.47 | 4.75 | 93.97 |
| 7 | 0.39 | 3.86 | 97.83 |
| 8 | 0.17 | 1.74 | 99.57 |
| Variables | Component 1 | Component 2 | Component 3 |
| TL | 0.415 | 0.278 |  |
| TD | 0.381 |  | -0.271 |
| LA | 0.106 | -0.549 | -0.292 |
| LM | 0.298 | -0.446 | -0.225 |
| SLA | -0.308 | -0.342 |  |
| NP | -0.383 |  | -0.316 |
| *k* |  | 0.434 | -0.512 |
| SS |  | -0.297 | 0.494 |
| SD | 0.336 |  | 0.409 |
| LI | -0.469 | 0.127 | 0.105 |


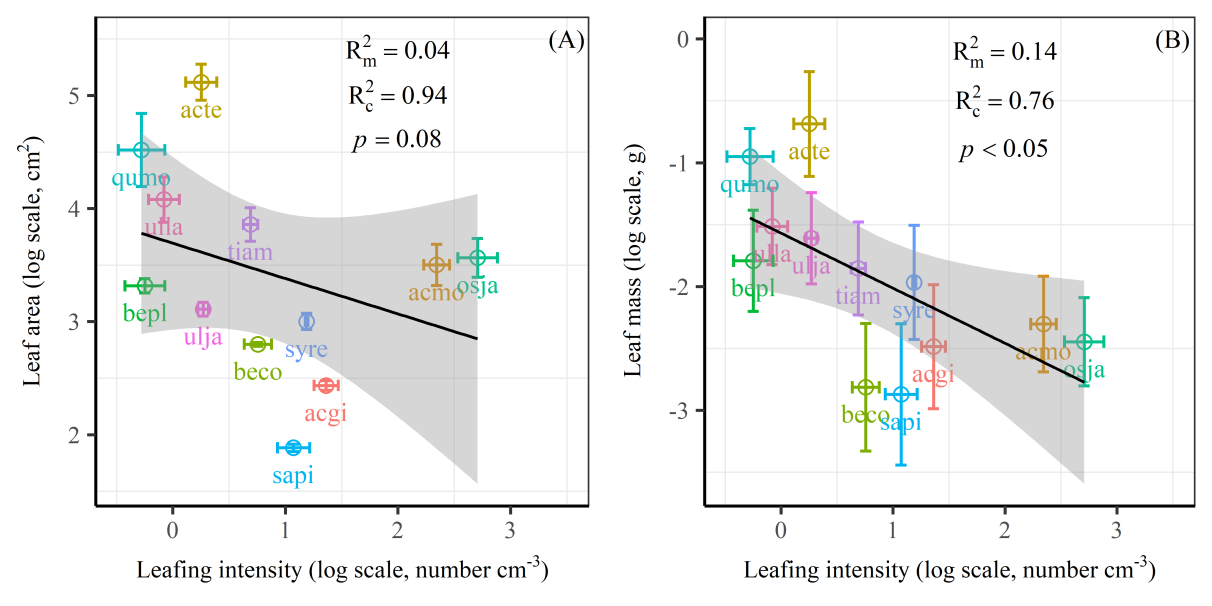
Figure S1 Relationships of volume-based leafing intensity with individual leaf area (A) and individual leaf mass for 12 simple-leaved tree species (B) from linear mixed models with leafing intensity as the mixed factor and tree species as the random factor. Marginal R^2^ ($R_{m}^{2}$) reflects the proportion of the variance explained by fixed factors and conditional R^2^ ($R_{c}^{2}$) reflects the proportion of the variance explained by both fixed and random factors. Average value per tree species and its 0.1-fold standard error were given. Grey bands show 95% confidence intervals. The abbreviations of tree species were provided in Table 1.


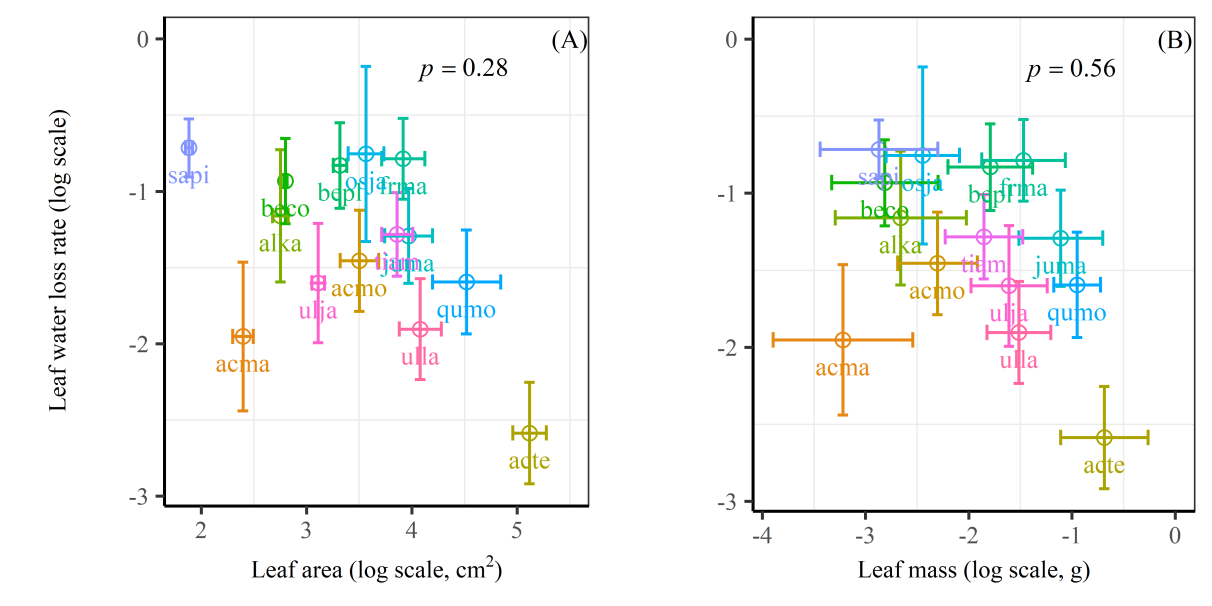


Figure S2 Relationships of 10 simple-leaved and 4 compound-leaved tree species leaf water loss rate (*k*) with individual leaf area (A) and individual leaf mass (B) from linear mixed models with individual leaf area and individual leaf mass as the mixed factor and tree species as the random factor. Marginal R^2^ ($R_{m}^{2}$) reflects the proportion of the variance explained by fixed factors and conditional R^2^ ($R_{c}^{2}$) reflects the proportion of the variance explained by both fixed and random factors. Average value per tree species and its 0.1-fold standard error were given. Grey bands show 95% confidence intervals. The abbreviations of tree species were shown in Table 1.
